# Probiotic *Bacillus cereus* Strains, a Potential Risk for Public Health in China

**DOI:** 10.3389/fmicb.2016.00718

**Published:** 2016-05-23

**Authors:** Kui Zhu, Christina S. Hölzel, Yifang Cui, Ricarda Mayer, Yang Wang, Richard Dietrich, Andrea Didier, Rupert Bassitta, Erwin Märtlbauer, Shuangyang Ding

**Affiliations:** ^1^Beijing Advanced Innovation Center for Food Nutrition and Human Health, College of Veterinary Medicine, China Agricultural UniversityBeijing, China; ^2^Department of Veterinary Sciences, Ludwig Maximilian University of MunichOberschleißheim, Germany; ^3^National Center for Veterinary Drug Safety Evaluation, China Agricultural UniversityBeijing, China

**Keywords:** *Bacillus cereus*, probiotic, enterotoxin, antimicrobial resistance, China, tetracycline resistance gene *tet*(45), site-specific recombination, dif site

## Abstract

*Bacillus cereus* is an important cause of foodborne infectious disease and food poisoning. However, *B. cereus* has also been used as a probiotic in human medicine and livestock production, with low standards of safety assessment. In this study, we evaluated the safety of 15 commercial probiotic *B. cereus* preparations from China in terms of mislabeling, toxin production, and transferable antimicrobial resistance. Most preparations were incorrectly labeled, as they contained additional bacterial species; one product did not contain viable *B. cereus* at all. In total, 18 *B. cereus* group strains—specifically *B. cereus* and *Bacillus thuringiensis—*were isolated. Enterotoxin genes *nhe, hbl, and cytK1*, as well as the *ces*-gene were assessed by PCR. Enterotoxin production and cytotoxicity were confirmed by ELISA and cell culture assays, respectively. All isolated *B. cereus* group strains produced the enterotoxin Nhe; 15 strains additionally produced Hbl. Antimicrobial resistance was assessed by microdilution; resistance genes were detected by PCR and further characterized by sequencing, transformation and conjugation assays. Nearly half of the strains harbored the antimicrobial resistance gene *tet*(45). In one strain, *tet*(45) was situated on a mobile genetic element—encoding a site-specific recombination mechanism—and was transferable to *Staphylococcus aureus* and *Bacillus subtilis* by electro-transformation. In view of the wide and uncontrolled use of these products, stricter regulations for safety assessment, including determination of virulence factors and transferable antimicrobial resistance genes, are urgently needed.

## Introduction

*Bacillus cereus* is a common cause of foodborne infectious disease: in a 10-year US survey *B. cereus* was ranked the fifth most common bacterial species causing foodborne outbreaks (http://www.cdc.gov/foodsafety/pdfs/fdoss-98-08-appendix-contributing-factors-508c.pdf; for detailed outbreak analysis see Bennett et al., 2013). Worldwide, Chinese food is frequently associated with foodborne *B. cereus* outbreaks (Notermans and Batt, 1998). Accordingly, *B. cereus* accounted for 13.4% of 1082 bacterial foodborne outbreaks between 1994 and 2005 in China; *B. cereus* was ranked as the second after *Salmonella* spp. amongst the most frequent bacterial causes of foodborne disease in Chinese inland provinces (Wang et al., 2007). This might be partly attributed to consumption habits, since *B. cereus* outbreaks are regularly associated with the consumption of rice (Ankolekar et al., 2009). In addition to rice (and pasta), meat, and dairy products are also important sources of foodborne *B. cereus* outbreaks in humans (Ruusunen et al., 2013; López et al., 2015). There are two different types of foodborne disease related to *B. cereus*: intoxication due to pre-formed cereulide or infection, followed by intra-intestinal production of enterotoxins such as Nhe and Hbl (Stenfors Arnesen et al., 2008). Foodborne *B. cereus* disease is generally self-limiting, although severe cases and even deaths have been reported (Bottone, 2010).

Though, being known as an important foodborne pathogen, *B. cereus* is licensed as a probiotic for men and livestock. In China, the safety of *B. cereus* probiotics is assessed and regulated; however, these regulations are partly rendered obsolete by recent research on *B. cereus*, particularly the identification of new virulence factors (Kim et al., 2015; Omer et al., 2015; Kilcullen et al., 2016). Thus, there is a lack of appropriate safety assessment for these probiotics in China—and also in other parts of the world—although, probiotics are even used to treat bacterial infections (Isolauri, 2003). In legal terms, probiotics are usually considered as supplements or foods—a fact which is critically discussed (Arnold, 2013). Apart from one pseudo-outbreak (Kniehl et al., 2003), no case of foodborne *B. cereus* infection due to the use of probiotics has theretofore been reported. However, a fatal infection following the application of a probiotic *Bacillus subtilis* preparation was reported in an immune-compromised patient (Oggioni et al., 1998).

Probiotic strains are applied to livestock as well. Thus, the use of *B. cereus* probiotics in livestock production might provide an additional hazard for human health. In China, animal wastes are traditionally used as feed in fish ponds; this creates a melting pot for bacteria of human, animal, and environmental origin and thereby facilitates horizontal gene transfer (Allen et al., 2010). Thus, besides the lack of detectable toxicity, future probiotic safety regulations should take into account the potential of transferring antibiotic resistance genes.

We hypothesize that toxin production, transferable antimicrobial resistance, and contamination with other bacteria are important safety aspects of probiotics, but insufficiently assessed up to now. Therefore, we intended to re-evaluate these important safety parameters in commercial probiotic *B. cereus* products.

## Materials and methods

### Isolation and identification of bacteria

Products were collected between 2013 and 2015 from nine provinces in China (Table 1). This investigation included all four *B. cereus* preparations licensed for human use. From the original packaging, the capsules with the dried probiotic preparations were removed with a tweezer, dipped into 80% ethanol, flame-treated and soaked in sterile distilled water until the capsules where dissolved. Subsequently, the content was suspended and 0.1 ml of the suspension was plated on Columbia agar with 5% sheep blood (Oxoid, Wesel, Germany). Single colonies of each different phenotype were passaged on new agar plates. Matrix assisted laser desorption/ionization (MALDI-TOF) mass spectrometry was used for bacterial identification (Schulthess et al., 2014). Phenotypic identification by means of biochemical tests with the API 50 CH and 20 E kits (BioMerieux) were used to further confirm and/or characterize the *B. cereus* group bacteria. The total DNA of each isolate was extracted from overnight cultures in casein hydrolysate-yeast medium plus 1% glucose (CGY) using the DNeasy blood and tissue kit (Qiagen, Germany), according to the protocol for Gram-positive bacteria. An extended multiplex PCR assay with five pairs of universal primers was used to identify the *cry*-group genes in the *Bacillus thuringiensis* strains (Ben-Dov et al., 1997).

**Table 1:** **Characterization of commercial ***B. cereus*** containing probiotic products in China**.

**Product No**.	**Strain No**.	**Origin**	**Label**	**Other species**	**Intended for use in**	**Species**	**Toxin type**
1	1	Jiangsu	mono	none	Human	*B. cereus*	Nhe, Hbl
2	2a	Jiangsu	mono	none^#^		*B. cereus*	Nhe, Hbl
	2b					*B. cereus*	Nhe
3	3	Henan	mono	none		*B. cereus*	Nhe, Hbl
4^.^	4	Zhejiang	mixed	not determined		*B. cereus*	Nhe, Hbl
5	5c	Jiangsu	mono	other *Bacillus* spp.	Animal	*B. cereus*	Nhe
6	6f	Hebei	mono	other *Bacillus* spp. *Acinetobacter* sp. *Enterococcus* sp. no ID.		*B. cereus*	Nhe, Hbl
7	7d	Hebei	mono	other *Bacillus* spp.		*B. thuringiensis*	Nhe, Hbl, Cry
8	8h	Hebei	mixed	other *Bacillus* spp.		*B. cereus*	Nhe, Hbl
9	9b	Hebei	mixed	other *Bacillus* spp. *Staphylococcus* sp. *Pseudomonas* sp.		*B. thuringiensis*	Nhe, Hbl, Cry
	9i					*B. cereus*	Nhe
10		Hebei	mixed	other *Bacillus* spp. *Enterococcus* sp. *Cronobacter* sp. *Acinetobacter* sp.		none	
11	11	Shandong	mono	none		*B. cereus*	Nhe, Hbl
12	12c	Jiangxi	mixed	other *Bacillus* spp.		*B. cereus*	Nhe, Hbl
13	13d	Guangdong	mono	other *Bacillus* spp.		*B. cereus*	Nhe, Hbl
14	14e	Shanxi	mixed	other *Bacillus* spp.	Plant	*B. cereus*	Nhe, Hbl
	14f					*B. thuringiensis*	Nhe, Hbl, Cry
15	15a	Hubei	mixed	other *Bacillus* spp.		*B. thuringiensis*	Nhe, Hbl, Cry
	15d					*B. cereus*	Nhe, Hbl

“*Toxin type” refers to the detection of the corresponding gene in the PCR-assay plus positive ELISA-reaction at the level of the reference strains plus cytotoxicity for Vero-cells*.

# *two different B. cereus strains*.

### Detection of enterotoxins and emetic toxin

The four main toxin genes of *B. cereus, ces, nhe, hbl*, and *cytK1*, were detected as previously described (Wehrle et al., 2009). Enzyme-linked immunosorbent assays were used to detect the production of *B. cereus* toxins, based on monoclonal antibodies specifically recognizing different epitopes of each toxin component (Zhu K. et al., 2013). Cytotoxicity assays were performed on Vero and HEp-2 cells to evaluate the toxic potential of the strains due to the production of enterotoxins (Nhe, Hbl, and CytK1) and/or emetic toxin (cereulide), respectively (Wehrle et al., 2009). *B. cereus* NVH 75/95, DSMZ 4384, and MHI 165 were used as reference strains for Nhe, Hbl, and cereulide, respectively (Wehrle et al., 2009; Lindbäck et al., 2010). *B. cereus* NVH 391/98 served as reference strain for CytK1, with a variant *nhe*-gene and no expression of the Nhe complex in the bacterial culture medium (Fagerlund et al., 2007).

### Assessment of phenotypic antimicrobial resistance

Antimicrobial resistance was assessed by a standardized microdilution assay (ISO 20776-1:2006) in cation adjusted Mueller Hinton broth (CAMHB). The assay included ampicillin, amoxicillin + clavulanate, cefaclor, cefoxitin, cefuroxime, ciprofloxacin, clindamycin, colistin, daptomycin, doxycycline, enrofloxacin, ertapenem, erythromycin, florfenicol, fosfomycin, gentamicin, imipenem, linezolid, meropenem, oxacillin, penicillin, quinupristin + dalfopristin, rifampicin, streptomycin, teicoplanin, telithromycin, tobramycin, and vancomycin.

### Detection of antimicrobial resistance genes

The presence of the tetracycline resistance genes *tet*(A), *tet*(B), *tet*(C), *tet*(D), *tet*(K), *tet*(L)/*tet*(45), *tet*(M), and *tet*(O) was assessed by end-point PCR as described elsewhere (Smith et al., 2004; Srinivasan et al., 2008; You et al., 2012); this selection included the most commonly detected *tet*-genes in *Bacillus cereus* (Agersø et al., 2002), but also four genes—*tet*(A), *tet*(B), *tet*(C), *tet*(D)—that were not yet found in the genus *Bacillus* (http://faculty.washington.edu/marilynr/tetweb3.pdf). PCR products were purified and submitted for sequencing (Sequiserve, Vaterstetten, Germany). The obtained sequences were compared with public databases (NCBI, EMBL-EBI) to verify the PCR products and to distinguish between *tet*(L) and *tet*(45) (You et al., 2013).

### Transferability of antimicrobial resistance

#### Transformation assay

##### Electro-transformation

The spread of antimicrobial resistance genes was determined in a modified transferability test as described elsewhere (Zhu W. et al., 2013). Plasmids of the *B. cereus* strain 9i were extracted by QIAfilter Plasmid Midi Kit (QIAGEN®). Subsequently, 500 ng of the purified plasmids were transformed into *Staphylococcus aureus* RN4220 electrocompetent cells, at 200 Ω, 25 μ Fd, 2500 V. After incubation at 37°C for 2 h, the resulting transformants were spread on brain heart infusion (BHI) agar plates containing 16 mg/L tetracycline, and incubated at 37°C for another 24 to 48 h. Isolated single colonies were tested for the presences of *tet*(45) by PCR assay, and the obtained PCR products were submitted for sequence analysis. Electro-transformation of *B. subtilis* ATCC 6051, *Escherichia faecalis* JH2-2, *Bacillus licheniformis* ATCC 14580 and *Bacillus amyloliquefaciens* ATCC 23842 followed the same protocol.

##### Chemical transformation

For the preparation of competent cells of *B. subtilis* ATCC 6051, *B. subtilis* was cultured on LB agar plates at 37°C for 24 h; a single colony was inoculated in 5 mL GM1 medium and shaken at 100 rpm (37°C) for 16 h. Subsequently, 2 mL of *B. subtilis* culture was transferred to 18 mL GM1 medium and shaken at 100 rpm (37°C) for another 3 h. Ten milliliters of the culture was subsequently transferred into 90 mL GM2 medium and shaken at 100 rpm (37°C) for 2 h. Lastly, all the cells were collected after centrifugation at 4000 rpm (4°C) for 10 min and re-suspended by 10 mL GM2 medium with the addition of 5 mL 30% glycerol to prepare the competent cells of *B. subtilis*. Chemical transformation was performed with *Escherichia coli* (DH5α) and *B. subtilis* competent cells as recipients using 0.5–1 μg plasmid DNA extracted from the donor strain *B. cereus* 9i, according to the manufacturer's instruction (TaKaRa).

#### Conjugation assay

Filter matings were performed with *E. coli* (EC600, rifampin resistant), *S. aureus* (RN4220), *E. faecalis* (JH2-2, rifampin resistant), and *B. subtilis* (ATCC 6051) as the recipient strains, following the method described elsewhere (Huys et al., 2004). Overnight cultures of the donor (*B. cereus* 9i) or recipient strains were diluted to 10^8^ CFU/mL in brain heart infusion (BHI) broth (Land Bridge Technology). Five microliters of each dilution was added into 1 mL BHI broth and incubated at 37°C for 4 h. The cultures of donor (20 μL) and recipients (60 μL) were mixed and filtered through a sterile 0.2 μm membrane filter (Millipore), and placed at 37°C for overnight incubation. After mating, the colonies were washed with 1 mL BHI broth and plated on BHI agars, containing 8 μg/mL TET and further incubated at 37°C for 24 h. For the selection of potential transconjugants, the agar plates contained 8 μg TET /mL; for *E. coli* EC600 and *E. faecalis* JH2-2, 25 μg rifampin /mL was added. Potential transformants grown on the selective agar plates were identified by 16S rRNA-gene sequences and tested for the presences of *tet*(45) by PCR assay.

#### Genetic environment of *tet*(45)

The DNA of *B. cereus* 9i was extracted (Wizard Genomic DNA Purification Kit, Promega) and partially identified by high-throughput sequencing (Illumina Hiseq 2500, performed at BerryGenomics Inc., Beijing China). A preliminary draft assembly of the genomic fragments was conducted using CLC Genomics Workbench 5 (CLC Bio, Aarhus, Denmark).

Once the surrounding of *tet*(45) was known, we designed primer pairs targeting the upstream- and downstream-environment of *tet*(45): UP-*tet*(45) (fw: TGGTTGAAGAGGCAC TCTATGG, rv: GGCGTAAATTTGACTGTGAATGA), *tet*(45)-INT1 (fw: TCATCGTCA TTAGCTGGTTGGT, rv: AAGTTGCAGAGGACATGGAAAAAC), INT1-INT2 (fw: TTT TCGGGGTTGATGGGCAA, rv: CGATGAGGAAAAACATCGAGAATCA), INT2-HTH (fw: GCGTGGCTTGTCCTTTCTAAC, rv: AGAACCCCAACAAGACTCCC), in order to verify the environment of *tet*(45) in representatives of each different variant of *tet*(45). All PCR-reactions were performed with an initial denaturation of 5 min at 94°C, 35 cycles of 60 s at 94°C, 60 s at 54°C, 90 s at 72°C, and a final elongation of 5 min at 72°C. *B. cereus* 9i served as positive control.

## Results

### Presence of multiple bacterial species

A total of 66 morphologically different strains were isolated from 15 typical samples labeled as products containing *B. cereus* in mono-preparation (Product No. 1–3, 5–7, 11, and 13) or mixed preparation (Product No. 4, 8–10, 12, 14, and 15). Based on bacterial morphology and biochemical testing results, as well as *cry*-gene based multiplex-PCR and MALDI-TOF mass spectrometry, these 66 isolates were classified as belonging to more than 10 different bacterial species; 18 strains were identified as belonging to the *B. cereus* group (Table 1). Four of these strains were further identified as *B. thuringiensis* due to the presence of the plasmid-borne *cry*-genes encoding insecticidal crystal toxins (*cry*1+*cry*2+*cry*7/8 in strains 9b, 14f, and 15a; *cry*2 in strain 7d). One sample (No. 10) did not contain viable *B. cereus*, but viable *B. subtilis* spores, instead. In total, this product contained nine different viable bacterial strains, including *Cronobacter sakazakii* and *Acinetobacter baumannii*.

### Detection of toxins

The PCR-assay detected *nhe* in all isolated *B. cereus* group strains (Table 1); *hbl* was detected in 15 isolates; accordingly, all strains were cytotoxic for Vero-cells. None of the isolates harbored *ces*-genes which are a hallmark of emetic strains, or *cyt*K1. Enzyme-linked immunosorbent assays confirmed that non-hemolytic enterotoxin (Nhe) and hemolysin BL (Hbl) were expressed by the *nhe*-/*hbl*-positive strains at the levels of the respective positive controls.

### Antimicrobial resistance

**Figure 1:**
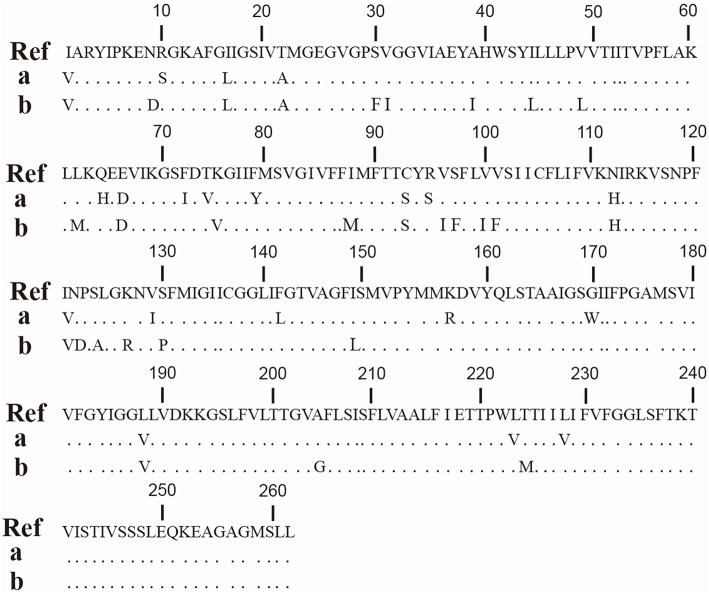
**Partial amino acid sequence of Tet(45)**. Amino acid sequence alignment of *tet*(45)-amplicons (**a**, strain No. 6f, 7d, 8h, 11, 13d, 14e and 15d) and the mobile genetic element- *tet*(45)-amplicon (**b**, strain No. 9i) with Tet(45) from *Bhargavaea* spp. (**Ref**, You et al., 2013; blastx, https://npsa-prabi.ibcp.fr).

Microdilution according to ISO-20776 indicated natural (species-specific) insusceptibility to penicillins (ampicillin, oxacillin, penicillin), cephalosporins (cefaclor, cefoxitin, cefuroxime), and colistin, as well as varying susceptibility for daptomycin, amoxicillin+clavulanate, and fosfomycin (Table 2). Microdilution further revealed eight isolates (44%) with elevated MIC-values for doxycycline (1–4 mg/L) whereas all other isolates had MIC-values ≤ 0.125–0.25 mg doxycycline /L. The same eight strains were positive for *tet*(45). Sequence analysis revealed that seven *tet*(45) amplicons were identical among each other, but the amplicon of the eighth strain, 9i, showed a different sequence (Figure 1).

**Table 2:** **Minimum inhibitory concentrations (MICs, mg/l) of 22 antimicrobials and presence /absence of ***tet***(45) in probiotic ***Bacillus cereus*** / ***Bacillus thuringiensis*** strains**.

**No**.	**Intended for use in**	**Species**	***tet*(45)**	**AMC**	**CIP**	**CLI**	**DPT**	**DOX**	**ENR**	**ERT**	**ERY**	**FLL**	**FOS**	**GEN**	**IMP**	**LEV**	**LIZ**	**MER**	**QDA**	**RIF**	**STR**	**TEI**	**TEL**	**TOB**	**VAN**
1	Human	*B. cereus*		>64	0.125	4	2	0.25	0.125	≤0.25	2	2	16	0.5	1	0.125	2	≤0.25	0.5	0.5	8	0.5	0.063	0.5	1
2a		*B. cereus*		>64	0.125	2	>4	≤0.125	0.125	≤0.25	1	1	16	1	1	0.125	1	1	1	0.5	8	0.5	0.063	≤0.25	1
2b		*B. cereus*		8	0.25	1	0.5	≤0.125	0.125	≤0.25	0.5	1	16	1	0.25	0.125	1	≤0.25	0.5	0.5	8	0.25	≤0.031	1	0.5
3		*B. cereus*		> 64	0.125	2	2	≤0.125	0.125	≤0.25	1	1	16	1	1		1	0.5	0.5	0.5	8	0.5	0.063	0.5	1
5c	Animal	*B. cereus*		4	0.25	1	>4	≤0.125	0.125	≤0.25	8	1	16	0.5	0.25	0.25	2	≤0.25	1	0.5	8	0.25	0.5	≤0.25	1
6f		*B. cereus*	+	>64	0.25	4	4	2	0.25	0.5	1	1	16	0.5	1	0.125	1	0.5	1	0.5	8	0.5	0.063	1	1
7d		*B. thuringiensis*	+	>64	0.25	2	4	2	0.125	≤0.25	1	1	16	1	1	0.25	1	1	1	0.5	8	0.5	≤0.031	0.5	1
8h		*B. cereus*	+	>64	0.125	2	4	2	0.125	≤0.25	1	1	16	1	1	0.125	1	≤0.25	1	0.5	8	0.5	0.063	0.5	1
9b		*B. thuringiensis*		>64	0.125	1	4	≤0.125	0.125	≤0.25	0.5	≤0.5	64	2	1	0.125	1	1	1	0.5	8	0.25	≤0.031	1	1
9i		*B. cereus*	+	64	0.5	2	>4	4	0.25	≤0.25	1	1	64	0.5	0.25	0.25	2	≤0.25	1	1	8	1	≤0.031	0.5	2
11		*B. cereus*	+	>64	0.25	2	>4	2	0.125	≤0.25	1	1	16	0.5	1	0.125	1	≤0.25	1	0.5	8	0.5	0.063	0.5	1
12c		*B. cereus*		64	0.125	2	2	≤0.125	0.125	0.5	1	1	16	0.5	2	0.125	1	0.5	0.5	0.5	8	0.5	0.125	≤0.25	1
13d		*B. cereus*	+	>64	0.125	4	4	2	0.25	≤0.25	1	1	16	0.5	1	0.125	1	0.5	1	0.5	8	0.5	≤0.031	1	1
14e	Plant	*B. cereus*	+	32	0.25	2	4	1	0.125	≤0.25	1	1	16	0.5	0.5	0.125	1	≤0.25	1	0.5	8	0.5	≤0.031	0.5	1
14f		*B. thuringiensis*		>64	0.125	1	>4	≤0.125	0.125	≤0.25	0.5	1	32	1	1	0.125	1	0.5	0.5	0.5	8	0.25	≤0.031	1	1
15a		*B. thuringiensis*		>64	0.125	1	4	≤0.125	0.125	≤0.25	0.5	1	128	1	1	0.125	1	≤0.25	1	0.5	8	0.25	≤0.031	1	1
15d		*B. cereus*	+	64	0.125	2	4	1	0.125	≤0.25	1	1	16	1	1	0.125	1	≤0.25	1	0.5	8	0.5	≤0.031	1	1
*B. cereus* ATCC 10876			n.d.	32	0.25	1	n.d.	0.5	0.125	≤0.125	0.5	1	n.d.	0.5	4	0.125	1	≤0.063	0.5	n.d.	n.d.	n.d.	0.063	n.d.	1

*MICs were determined according to ISO-20776. All isolates where negative for tet(M), tet(K), tet(O), tet(A), tet(B), tet(C), tet(D). All tet(L)/tet(45)-amplicons revealed higher sequence identity with tet(45) than with tet(L)*.

AMC, amoxicillin + clavulanate; CIP, ciprofloxacin; CLI clindamycin, DPT, daptomycin; DOX, doxycycline; ENR, enrofloxacin; ERT, ertapenem;

ERY, erythromycin; FLL, florfenicol; FOS, fosfomycin; GEN, gentamicin; IMP, imipenem; LEV, levofloxacin; LIZ, linezolid; MER, meropenem;

*QDA, qinupristin + dalfopristin; RIF, rifampicin; STR, streptomycin; TEI, teicoplain; TEL, telithromycin; TOB, tobramycin; VAN, vancomycin*.

### Plasmid extraction and horizontal transfer

**Figure 2:**
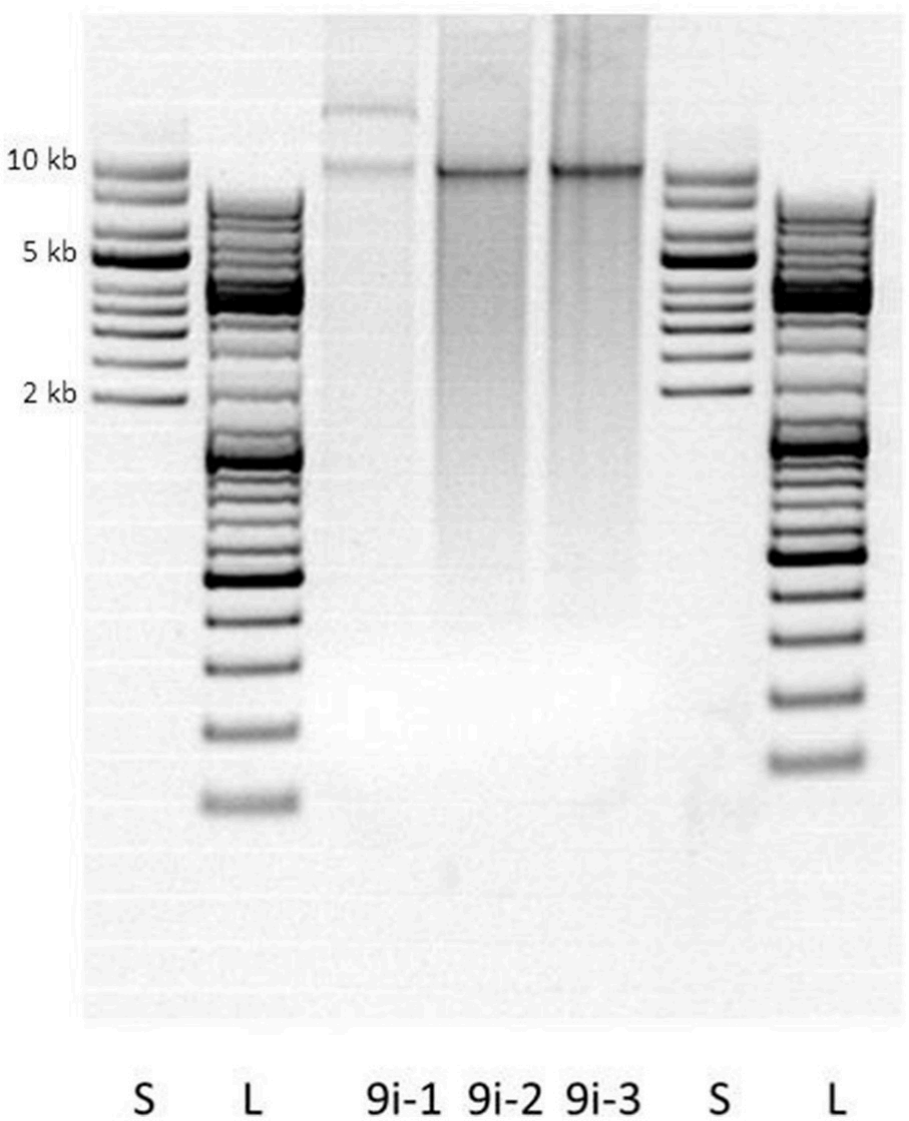
**Agarose gel electrophoresis after plasmid extraction of ***B. cereus*** 9i**. 1–3: three independent replicates. S supercoiled ladder, L linear ladder. Note: two putative plasmid bands were visible in a second agarose gel electrophoresis of extract 9i-3, at the same height as in extract 9i-1.

Further investigations demonstrated that *B. cereus* 9i contained two plasmids, one of ~10 kB and one of > 50 kB in size (Figure 2). Both plasmids were assumedly low copy plasmids, generating reproducible, but weak bands on agarose gel (Figure 2). The *tet*(45)-gene of 9i was successfully transferred into *S. aureus* RN4220 and *B. subtilis* ATCC6051 (but not *E. coli*) by electro-transformation. Transformed *S. aureus* and *B. subtilis* increased their doxycycline MIC-values from ≤ 0.25 to 2 mg/L. After electro-transformation, the *tet*(45)-gene was amplified in the DNA-extracts of the putative transformants. However, neither chemical transformation nor conjugation assays resulted in detectable transfer of *tet*(45).

### Genetic environment of *tet*(45)

**Figure 3:**
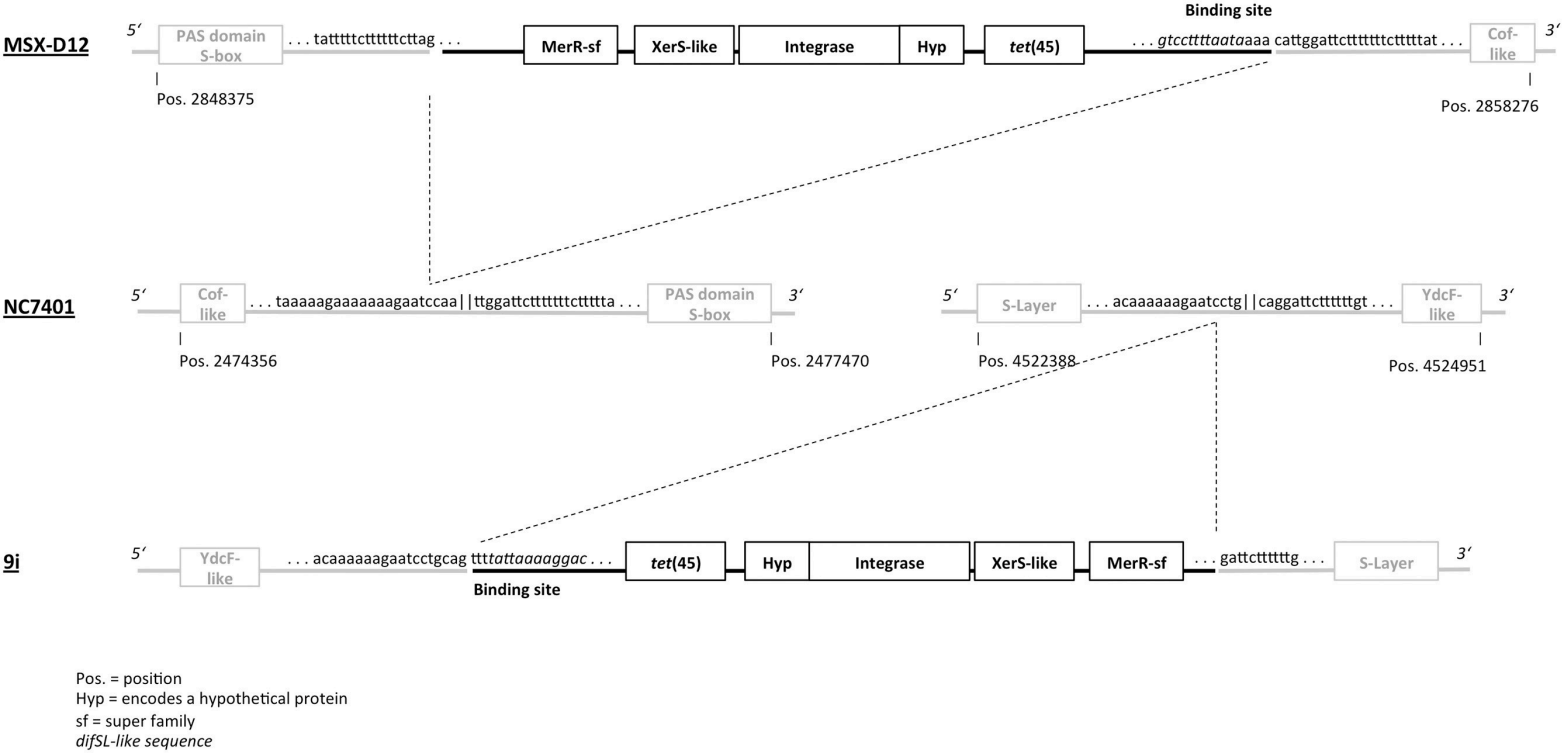
**Genetic environment of ***tet***(45) in ***B. cereus*** 9i**. Surrounding sequences of *tet*(45) in *B. cereus* 9i (this study) and *B. cereus* MSX-D12 (Timmery et al., 2011) are shown relatively to *B. cereus* NC7401 (AP007209.1). Note: most Genbank entries for *Bacillus cereus* show the same 5'-3'-orientation of the YdcF-like-/ S-layer protein encoding sequences and the Pas domain S-box-/Cof-like protein encoding sequences as 9i and MSX-D12, respectively, not as NC7401. NC7401 was chosen as reference sequence since the palindromic sequence between the Pas domain S-box-/Cof-like protein encoding sequences is conserved here, but not in the *Bacillus cereus* reference genomes.

Partial sequence analysis of the DNA extracts is visualized in Figure 3. The *tet*(45)-gene is located on a putatively mobile genetic element (MGE) of 6789 bp (Genbank accession number KX091844). Upstream, the MGE starts with a *dif* SL-like binding motif (Figure 3) situated on a 413 bp sequence of unknown function, which precedes *tet*(45). Downstream, *tet*(45) is followed by a gene encoding a protein of unknown function. The insertion element further contains two different integrase genes (2181/1347 bp)—at least one of the integrases with significant similarity to a site-specific tyrosine recombinase, XerS—and a 552 bp gene encoding a MerR superfamily protein (putative excisionase) with a helix-turn-helix domain.

With DNA-extracts of two representatives of the second *tet*(45)-variant, no amplicons were generated when we used primer pairs UP-*tet*(45), *tet*(45)-INT1, INT1-INT2, or INT2-HTH, while amplicons of the expected size were found for *B. cereus* 9i (positive control) in each PCR-reaction.

Sequences of the mobile genetic element and of the second *tet*(45) variant (partial sequence) were deposited in Genbank (accession numbers KX091844 and KX091845).

## Discussion

### Mislabeled probiotic products

Five products labeled as *B. cereus*-monopreparations contained bacterial species which did not belong to the *B. cereus* group. Mislabeling of probiotic *Bacillus* strains is a common problem also observed in Europe, where Bactisubtil has been firstly labeled to contain *B. subtilis* spores instead of *B. cereus* spores (Huys et al., 2013). Vice versa, in our study one preparation for use in livestock did not contain any viable *B. cereus* group strain, but *B. subtilis* spores, instead. In addition, the product contained two opportunistic human pathogens, *A. baumannii* and *C. sakazakii*; the latter outnumbered the *B. subtilis* spores (data not shown).

### Toxins

Due to the presence of plasmid-borne *cry*-group-genes, which encode insecticidal toxins, four strains were identified as *B. thuringiensis*. Two of them were isolated from preparations that were not labeled to contain *B. thuringiensis*. *B*. *cereus* is closely related to *B. thuringiensis*: the 16S rRNA-gene sequences share more than 99% similarity (Stenfors Arnesen et al., 2008). The insecticidal toxins of *B. thuringinesis* are not harmful for mammals; however, the isolates also produced enterotoxins (Hbl and Nhe), as did all of the *B. cereus* group strains (Table 1). Hbl and Nhe are pore-forming toxins, each composed of three individual proteins, which show a specific order of binding to mammalian cells. Although the mode of action of these toxins is not fully understood, assembly of the components seems to occur partly in solution and is completed on the cell surface resulting in pore formation and cell lysis (Lindbäck et al., 2010; Sastalla et al., 2013). For both toxins, the presence of all three compounds is necessary to evoke toxicity, and only strains expressing complete Hbl and/or Nhe can be regarded as diarrheic (Stenfors Arnesen et al., 2008). This was the case for all isolated *B. cereus* group strains, the expression level of Nhe was even similar to the reference strain isolated from a food poisoning case in Norway (Lund and Granum, 1996; data not shown). A probiotic *B. cereus* strain licensed in the European Union has also been shown to produce biologically active enterotoxins (Nhe, Hbl; Duc et al., 2004). Interestingly, a preparation of this strain was withdrawn for use in animals, while still included in a licensed—but originally mislabeled—preparation for human use (Huys et al., 2013; Hong and Cutting, 2005).

### Enteropathogenic *B. cereus*

The infectious dose of enteropathogenic *B. cereus* was proposed to be 1 × 10^5^ to 1 × 10^8^ CFU or spores/g (Granum and Lund, 1997). Variations in this infectious dose are attributed to strain traits, since strains differ both in the amount of toxins produced (Dommel et al., 2011; Jessberger et al., 2015; Lücking et al., 2015), and also in their capability for intestinal persistence. Probiotic strains are applied in a dose of 10^7^–10^9^CFU or spores/g; they have also been selected for their ability to colonize the intestinal tract (Duc et al., 2004). Thus, given their high likelihood to establish colonies in the intestine, unsafe probiotic strains might provide a major health risk, compared to strains casually ingested with food. Such health risks have been demonstrated in case studies. For example, a fatal infection with a probiotic *Bacillus* preparation in an immune-compromised patient has been reported (Oggioni et al., 1998).

### Transferable antimicrobial resistance gene

Besides the lack of toxicity, the presence of transferable resistance genes or not is another major safety parameter for probiotic strains. At least one of the strains investigated in the current study carried a tetracycline resistance gene, *tet*(45), on a mobile genetic element. In *Bacillus* spp., some corresponding entries in Genbank are labeled as “tetracycline resistance MFS efflux pump,” which overcomes the problem that the protein encoded by *tet*(45) in *Bacillus* spp. is similar to the product of *tet*(L) so that partial sequences might not allow unambiguous distinction. However, the complete sequence presented here has higher similarity to the reference sequences of *tet*(45) than to the reference sequences of *tet*(L) (http://faculty.washington.edu/marilynr/tetweb4.pdf).

The mobile element contained a *dif* SL-like binding site, the *tet*(45)-gene, one ORF encoding a hypothetical protein, two integrase genes (one of the XerS superfamily, but without significant similarity to the XerS-encoding gene of *Streptococcus suis*), two non-coding-sequences of unknown function and a gene encoding a MerC-superfamily protein (putative excisionase). The coherent mobile element as found here was not described in *Bacillus* spp. before, but NCBI-BLAST revealed 93% identity to an inverse sequence of *B. cereus* MSX-D12 (pos. 2856985–2851281), isolated 2005 in the Antarctic Concordia Station by Timmery et al. (2011). At 5′, the putative MGE in 9i begins with an adenin-rich-motif (caaaaaagaatcc). This motif was identified as the “left-hand” part of a putative Xer-binding/cleavage site, since the 3′ (“right-half”)-site (**tattaaaag**g**ac**ag) downstream the motif had remarkably high identity to *dif* SL (Leroux et al., 2011; bold = consensus), whereas the sequence had much lower similarity to known Xer-binding sites of *Bacillus* spp. (e.g., *cer, dif, dif* BS). MSX-D12 presented a complementary *dif* SL-like sequence at 3′ (Figure 3).

The sequence situated upstream of the putative MGE in 9i contains one single caaaaaagaatcctg-cag-motif; an inverted repeat of the motif, gattcttttttg (missing the cag-sequence which is adhered at the 5′ cleavage site), follows immediately downstream of the 3′ end of the MGE. In MSX-D12, the same—but undisrupted—sequence is situated ~2,100,000 bp upstream of the putative mobile element described here, forming a perfect 30 bp palindrome (caaaaaagaatcctg||caggattcttttttg). Interestingly, the both opposed ends of the palindrome are identical to the opposed ends of the “left-hand”-part of *dif* SL (Leroux et al., 2011).

Identical upstream and downstream genes—encoding a YdcF-like protein and an S-layer protein—are found in MSX-D12 (not shown), NC7401 and 9i (Figure 3, respectively) next to both ends of the palindromic cleavage site. This raises strong suspicion that, in 9i, *tet*(45) was inserted into this sequence by help of (and together with) the site-specific recombinases encoded downstream. In MSX-D12, the MGE was inserted at another palindromic site (Figure 3), was inversely orientated, and its insertion was accompanied by a crossover-event so that the palindromic sequence at both ends of the MGE was directly mirrored (tatttttctttttttcttag||gattcttttttctttttat) instead of being complimentary.

Remarkably, while the binding site had high homology with the *dif* SL-site identified in *Streptococcus* and *Lactococcus*, the XerS-like protein had no significant similarity with the XerS-proteins of *Streptococcus* or *Lactococcus*, and two different integrase genes were combined in the MGE, hinting toward a XerC/XerD-like dimeric process of site-specific recombination instead of a “stand-alone” mechanism as proposed for XerS (Leroux et al., 2011).

*B. cereus* 9i contained two plasmids. However, in the plasmid extracts we could neither identify any gene associated with conjugation, nor did we succeed in transferring *tet*(45) by means of conjugation assays. The YdcF-like-protein and the S-layer-protein which are connected by the putative insertion site are chromosomally encoded in *Bacillus* spp. This does not necessarily mean that *tet*(45) is chromosomally integrated in 9i, since chromosomal genes could also be transferred to plasmids, as vice versa. Very recently (2016-03-21) there was a new Genbank entry of a *B. thuringiensis* plasmid (pBT1850294, CP014284.1) which encodes a significantly similar pair of integrase genes (66% nucleotide identity). However, we assume that the transfer of *tet*(45) to *S. aureus* and *B. subtilis* was not necessarily associated with its presence on one of the two plasmids found in 9i, but was associated with a small mobile genetic element, instead. Such elements could be easily integrated into naturally competent hosts after natural transformation, especially if insertion relies on conserved integrases like the Xer-superfamily (Midonet and Barre, 2014), as indicated here. The *dif* SL-like “right-hand”-motif is present in the genome of a variety of other bacteria, e.g., *Lawsonia intracellularis* (e.g., CP004029.1), *Mycoplasma* spp. (e.g., CP014346.1), *Clostridia* spp. (e.g., CP011803.1), *A. baumannii* (e.g., CP014528.1), *Staphylococcus* spp. (e.g., KT780704), and other *Bacillus* spp. (e.g., CP012600.1). While it is unknown whether these bacteria express recombinase enzymes that bind there, at least streptococci and lactococci are known to do so. Moreover, *Aneurinibacillus soli* (AP017312.1) and *B. mycoides* (CP007626.1) encode a pair of integrases (and also a MerC-superfamily protein) with significant similarity to the recombinase enzymes described here, suggesting a further possibility for interspecific and even intergeneric transfer of the MGE.

The detection of a mobile *tet*(45)-gene in probiotics is certainly undesirable. Due to the presence of a tetracycline resistance gene located on a mobile genetic element, together with insufficient data on toxicity, the probiotic *B. cereus* strain of Esporafeed Plus has been considered unsafe for use as a feed additive for calves and pigs in Europe in 1999 (http://ec.europa.eu/food/fs/sc/scan/out39_en.pdf, accessed March 31, 2016); more recently, *Bacillus toyonensis* was considered unsafe for use in chickens, pigs, cattle and rabbits for the same reason (http://www.efsa.europa.eu/en/efsajournal/pub/3766, accessed May 12, 2016). The *tet*(45)-gene has been originally detected in an environmental bacterium, *Bhargavaea* spp., from which it was successfully transferred to *E. coli* (You et al., 2012). Many antibiotic resistance genes (ARGs) of common human pathogenic bacteria are assumed to originate from environmental bacterial communities (Martínez, 2008; Forsberg et al., 2012). As a spore-forming bacterium and original soil inhabitant, *B. cereus* has a distinct ability to persist in and (re-)adapt to the environment (Carlin et al., 2010). Therefore, it cannot be ruled out that mobile resistance determinants may be further transferred to other soil bacteria, but also to other animal or human pathogens after excretion of such probiotic *B. cereus* strains. The detection of *tet*(M)-carrying *B. cereus* in soil was directly related to manure application in the past (Agersø et al., 2002; Roberts, 2012). In view of the wide and uncontrolled use of probiotics, strict regulations are urgently needed to decrease the probability of transfer of ARGs and safety assessment of these products must include the determination of antimicrobial resistance profiles.

Here, we summarize the possible health hazards of commercial probiotic products advertised to contain *B. cereus* in China due to mislabeling, toxin production and the presence of transferable antimicrobial resistance. The growing presence of pharmaceutical and biotechnological companies has contributed to an increase of *B. cereus* related products, but these products are often of poor quality and incorrectly designated. It must be emphasized that the safety of these commercially available probiotics is not a limited local public health issue in China, but may also be relevant worldwide due to global trade and international travel.

## Author contributions

KZ, EM, and SD conceived the project. KZ, CH, YC, RM, YW, AD, RD, RB, and SD conceived and performed experiments and/or data analysis. CH, KZ, EM, and SD wrote the manuscript, RB conceived the graphical design. KZ and CH contributed equally to this work.

### Conflict of interest statement

The authors declare that the research was conducted in the absence of any commercial or financial relationships that could be construed as a potential conflict of interest.
